# SFTSV Infection Induced Interleukin-1β Secretion Through NLRP3 Inflammasome Activation

**DOI:** 10.3389/fimmu.2021.595140

**Published:** 2021-02-23

**Authors:** Jian-Wei Liu, Min Chu, Yong-jun Jiao, Chuan-Min Zhou, Rui Qi, Xue-jie Yu

**Affiliations:** ^1^ State Key Laboratory of Virology, School of Health Sciences, Wuhan University, Wuhan, China; ^2^ Institute of Pathogenic Microbiology, Jiangsu Provincial Center for Disease Prevention and Control, Nanjing, China

**Keywords:** severe fever with thrombocytopenia syndrome virus, pro-inflammatory cytokines, interleukin-1β, NLRP3 inflammasome, tick-borne virus

## Abstract

Severe fever with thrombocytopenia syndrome virus (SFTSV) is an emerging tick-borne virus that causes hemorrhagic fever. Previous studies showed that SFTSV-infected patients exhibited elevated levels of pro-inflammatory cytokines like interleukin-1β (IL-1β), indicating that SFTSV infection may activate inflammasomes. However, the detailed mechanism remains poorly understood. Herein, we found that SFTSV could stimulate the IL-1β secretion in the infected human peripheral blood mononuclear cells (PBMCs), human macrophages, and C57/BL6 mice. We demonstrate that the maturation and secretion of IL-1β during SFTSV infection is mediated by the nucleotide and oligomerization domain, leucine-rich repeat-containing protein family, pyrin-containing domain 3 (NLRP3) inflammasome. This process is dependent on protease caspase-1, a component of the NLRP3 inflammasome complex. For the first time, our study discovered the role of NLRP3 in response to SFTSV infection. This finding may lead to the development of novel drugs to impede the pathogenesis of SFTSV infection.

## Introduction

Severe fever with thrombocytopenia syndrome (SFTS) is an emerging hemorrhagic fever caused by SFTSV, a tick-borne virus. SFTSV is a segmented RNA virus that was first discovered in China in 2009 and has been reported in Japan, South Korea, and Vietnam (1–4). The major vector of SFTSV is *Haemaphysalis longicornis* tick that is native of East Asia and has been spread into Oceania and North America (5–7). SFTSV infection usually resulted in fever, thrombocytopenia, leukocytopenia, multiple organ failure with 20–30% case fatality rate (8). SFTS patients exhibited increased levels of pro-inflammatory cytokines, including interleukin 6 (IL-6), interleukin 10 (IL-10), and monocyte chemotactic protein 1 (MCP-1) (9). In the SFTS fatal cases, the patients exhibited even high levels of interleukin 1β (IL-1β), interleukin 8 (IL-8), and macrophage inflammatory proteins 1*α* and 1*β* than those of mild patients (10, 11). However, Song et al. reported that the SFTS fatal cases had elevated IL-6, TNF-α level and declined IFN-*β*, IL-1β level (12). The differences among these studies might be due to the patient cohorts used in the studies or the different time for the sera collection. But overall, SFTS patients had severe cytokine imbalance, especially in the fatal cases, which indicated that the inflammatory response may play an important role in the SFTS progress. However, the mechanism behind the secretion of these cytokines upon SFTSV infection has not been revealed.

Pro-inflammatory cytokines, including the IL-1 superfamily, are key mediators of the innate immune system, playing an important role in response to invading pathogens, including viruses, bacteria, and fungi (13). IL-1β, as an “administrator”, that arranges the following immune responses at both the local and systemic levels, stimulates macrophages to secret other inflammatory cytokines (14). During viral infection, the formation of IL-1β has two steps, priming and maturation. Upon the pathogen-associated molecular patterns (PAMPs) or damage-associated molecular patterns (DAMPs) are recognized by the pattern recognition receptors (PRRs), nuclear factor-*κ*B (NF-*κ*B) signaling cascade will be activated and promoted the transcription of pro-IL-1β. Then, Pro-IL-1β is cleaved by protease caspase-1 to mature into active IL-1β, which is a component of the inflammasome complex. Inflammasomes are cytoplasmic sensor/activators which show proteolysis functions in caspase-1 dependent manner, and especially target pro-IL-1β and pro-IL-18 to activate the cytokines (15). Among several inflammasomes, NLRP3 inflammasome is the best-characterized one and is linked to the maturation and release of IL-1β in viral infection (16–18). NLRP3 inflammasome comprises the sensor molecule NLRP3, the adaptor protein apoptosis-associated speck-like protein containing CARD (ASC), and the effector protease pro-caspase-1 (19). Upon activation, NLRP3 protein interacts with ASC and recruits pro-caspase-1 to form NLRP3-ASC-pro-caspase-1 complex, also named as NLRP3 inflammasome, which induces the pro-capase-1 autoproteolytic cleavage into the active form caspase-1. Activated caspase-1 leads to the cleavage and secretion of mature IL-1β and IL-18. Activation of the NLRP3 inflammasome and IL-1β release mediate host protection against various pathogen invasions. However, hyperactivation of the inflammasomes contributes to the pathogenesis of inflammatory diseases, such as lung injury in Severe Acute Respiratory Syndromes (SARS), viral encephalitis, and viral acute or chronic hepatitis (20–23).

Some small molecules have been shown to activate NLRP3 inflammasomes, like LPS, Nigericin, and ATP. LPS is capable of triggering a cascade of inflammatory processes through toll-like receptor 4 signaling pathway, which could further lead to more intricate biological responses including the secretion of a number of pro-inflammatory mediators like IL-1β (24). Nigericin and ATP can activate NLRP3 inflammasome by promoting potassium efflux, and they can enhance LPS induced inflammation. Also, some inhibitors, like CY-09 and VX-765, could prevent NRRP3 inflammasome activation. CY-09 directly binds to the ATP-binding motif of NLRP3 NACHT domain and inhibits NLRP3 ATPase activity, resulting in the suppression of NLRP3 inflammasome assembly and activation (25).

Previous studies demonstrated that SFTSV could infect many human cell lines including HUVECs, 2937, RD, Huh7, MEG-01, Jurkat, and THP-1 (26–28). Macrophages are the major immune cells that release pro-inflammatory cytokines including IL-1β. Jin et al. established an infectious model of SFTS in C57/BL6 mice which can result in hallmark symptoms of thrombocytopenia and leukocytopenia, and SFTSV replication was found in the mice spleen macrophages (27). Here, we found that SFTSV infection can stimulate the secretion of IL-1β in infected human peripheral blood mononuclear cells (PBMCs), human macrophages, and C57/BL6 mice sera. We demonstrate that the maturation and secretion of IL-1β during SFTSV infection is mediated by NLRP3 inflammasome activation. This process is dependent on protease caspase-1, a component of the NLRP3 inflammasome complex.

## Materials and Methods

### Reagents

Roswell Park Memorial Institute 1640 medium (RPMI 1640) and Dulbecco’s modified Eagle’s medium (DMEM) were obtained from Gibco (Grand Island, NY). Lipopolysaccharide (LPS), NLRP3 assembly inhibitor CY-09, and phorbol 12-myristate 13-acetate (PMA) were purchased from Sigma-Aldrich (St. Louis, MO). Nigericin and caspase-1 inhibitor VX-765 were purchased from InvivoGen (San Diego, CA). Monoclonal rabbit anti-human IL-1β (D3U3E) (1:1,000), monoclonal rabbit anti-human cleaved IL-1β (D3A3Z) (1:1,000), monoclonal rabbit anti-human NLRP3 (D2P5E) (1:1,000), and monoclonal rabbit anti-human caspase-1 (D7F10) (1:1,000) were purchased from Cell Signaling Technology (Beverly, MA). Monoclonal mouse anti-human ASC (sc-271054) (1:500) and polyclonal goat anti-human cleaved caspase-1 (sc-22163) were purchased from Santa Cruz Biotechnology (Santa Cruz, CA). Monoclonal mouse anti-*β*-actin (1:10,000) and HRP conjugated secondary antibodies including goat anti-human IgG, goat anti-rabbit IgG, and goat anti-mouse IgG were obtained from Proteintech Group, Inc (Rosemont, IL). FITC conjugated goat anti-human IgG secondary antibody (1:200) was obtained from Fitzgerald (Birmingham, WM, UK).

### Cells

PBMCs were isolated from peripheral blood by density gradient centrifugation. In brief, blood was diluted with an equal volume of RPMI 1640, and 8 ml diluted blood was gently layered over a 4 ml lymphocyte separation medium. The cells were centrifuged at 400*×g* for 20 min. Then, the cell layer was transferred to a new centrifuge tube and diluted with RPMI 1640. The remaining red blood cells were removed using a red blood cell lysis buffer (Sigma-Aldrich, St. Louis). The purified PBMCs were centrifuged at 300*×g* for 10 min and cultured in RPMI 1640 with 10% heat-inactivated fetal bovine serum (FBS; Gibco) (Grand Island, NY) at 37°C in 5% CO_2_ environment. After 4 h of incubation, the supernatants were aspirated and changed with a new cell culture medium.

THP-1 cells (a human monocytic cell line) were cultured in RPMI 1640 with 10% heat-inactivated FBS at 37°C in 5% CO_2_. THP-1 cells were stimulated to differentiate into macrophages by the addition of PMA (100 ng/ml). After 24 h, PMA was removed and the cells were cultured for another 24 h. THP-1 differentiated macrophages were infected with SFTSV at the indicated multiplicity of infection (MOI) for 2 h at 37°C or treated with LPS (1 μg/ml) for 4 h (or then 2 μM Nigericin for 30 min). After infection, the cells were gently washed three times with an appropriate cell culture medium and cultured with RPMI 1640 containing 2% heat-inactivated FBS. To inhibit the NLRP3 inflammasome assembly or caspase-1 activation, cells were treated with the indicated concentration of the NLRP3 assembly inhibitor CY-09 or caspase-1 inhibitor VX-765 for 1 h prior to the infection. After infection, cells were washed, and the inhibitor was added back to the cells with the medium.

HEK293T cells (a human embryonic kidney cell line) and Vero cells (an African green monkey kidney cell line) were maintained in DMEM supplemented with 10% heat-inactivated FBS at 37°C in 5% CO_2_.

### Virus

SFTSV (strain JS2011-013-1) was cultured on Vero cells with the DMEM medium containing 2% heat-inactivated FBS. The virus titer was determined by plaque assay. Briefly, Vero cells were cultured in a 12-well plate at a density of 2 × 10^5^ cells/well and infected with 200 μl 10-fold serially diluted virus solution for 2 h. Then the cells were washed and replenished with plaque medium supplemented with 1% carboxyl methylcellulose. The infected Vero cells were incubated for 7 days. After incubation, plaque medium was removed, and cells were fixed and stained with 4% formaldehyde and 0.5% crystal violet.

### Mice Infection

All mice used in our experiments are on a C57/BL6 genetic background, and all experiments were carried out with age and gender matched mice (4–5 weeks old, female). C57/BL6 mice were purchased from the Sanxia University (Yichang, China). Mice were infected with 2 × 10^6^ Pfu of SFTSV or the same volume PBS intraperitoneally (i.p.). The infected mice were sacrificed on 3 or 5 days post infection for collection of sera and spleens for analysis. For the analysis of murine samples, six mice were used in each group.

The animal study was approved by the ethics committee of medical school, Wuhan University (2019YF2013) and was conducted in accordance with the guidelines for the protection of animal subjects.

### Lentivirus Production and Infection

The targeting sequences of shRNAs for human NLRP3, ASC, and pro-caspase-1 are as follows: sh-NLRP3: 5′-GCGTTAGAAACACTTCAAGAA-3′; sh-ASC: 5′-GATGCGGAAGCTCTTCAGTTTCA-3′; and sh-pro-caspase-1: 5′-CACACGTCTTGCTCTCATTAT-3′. The shRNAs were inserted into the vector PLKO.1 to build the recombinant plasmids, which were then transfected into HEK293T cells together with the packaging plasmids psPAX2 and pMD2.G with Polyethyleneimine (PEI, Alfa Aesar) as described (17). The blank PLKO.1 vector was used as a negative control. Culture supernatants were harvested at 48 and 96 h after transfection and filtered through a 0.45 μm filter. The lentivirus was concentrated with a lentivirus concentration solution (25% PEG8000 with 750 mM NaCl) and resuspended in RPMI 1640. THP-1 differentiated macrophages were infected with the lentiviral particles in the presence of 5 μg/ml polybrene (Sigma) for 4 h. RPMI 1640 with 10% FBS (volume 1:1) was added and incubated for 24 h. Then the supernatants were aspirated and resuspended in a complete cell culture medium for an additional 48 h. The cells were lysed to detect each shRNA targeted protein by Western blot or infected with SFTSV.

To get the NLRP3-knockout THP-1 cells, CRISPR-Cas9 system was used and sgRNA (5′-GGGATGCAGCCCTTCTGGGG-3′) was inserted into the plasmid plentiCRISPR V2. The recombinant plasmid and the packaging plasmids (psPAX2 and pMD2.G) were co-transfected into HEK293T cells and the lentiviral particles were collected, and concentrated as described above. The suspended THP-1 cells were infected with the lentiviral particles in the presence of 5 μg/ml polybrene and screened with 2 μg/ml puromycin (Sigma) for three rounds.

### Quantitative PCR

Total RNA was extracted from the cells or the mice spleens with TRIzol reagent (Invitrogen) following the manufacturer’s instructions, and reversed to cDNA by High Capacity cDNA Reverse Transcription Kit (Thermo). Quantitative PCR was performed using the Roche LC480 and ChamQ SYBR qPCR Master Mix (Vazyme, Nanjing, China) in a 20 μl volume reaction mixture of 10 μl SYBR Green PCR master mix, 1 μl DNA diluted template, 0.5 μM forward and reverse primers, and RNase-free water. The sequences of quantitative PCR primers were provided in **Table 1**.

**Table 1:** Primers used for quantitative PCR analysis.

Genes	Forward primer (5′–3′)	Reverse primer (5′–3′)
human NLRP3	GATCTTCGCTGCGATCAACA	GGGATTCGAAACACGTGCATTA
human caspase-1	GCCTGTTCCTGTGATGTGGAG	TGCCCACAGACATTCATACAGTTTC
human ASC	AACCCAAGCAAGATGCGGAAG	TTAGGGCCTGGAGGAGCAAG
human IL-1β	CCAGGGACAGGATATGGAGCA	TTCAACACGCAGGACAGGTACAG
human β-actin	GACCACCTTCAACTCCATCAT	CCTGCTTGCTAATCCACATCT
mice NLRP3	TCACAACTCGCCCAAGGAGGAA	AAGAGACCACGGCAGAAGCTAG
mice IL-1β	ACTGTTTCTAATGCCTTCCC	ATGGTTTCTTGTGACCCTGA
mice β-actin	GGTGTGATGGTGGGAATGG	GCCCTCGTCACCCACATAGGA
SFTSV	GGGTCCCTGAAGGAGTTGTAAA	TGCCTTCACCAAGACTATCAATGT

### Enzyme-Linked Immunosorbent Assay

The concentrations of mature human IL-1β, IL-6, TNF-α, and mice IL-1β in supernatants or sera were measured according to manufactures’ instructions with the human IL-1β/IL-1F2 valukine™ ELISA Kit, human IL-6 valukine™ ELISA Kit, human TNF-α valukine™ ELISA Kit, and mouse IL-1β/IL-1F2 quantikine^®^ ELISA Kit. The four ELISA kits were purchased from R&D systems (Minneapolis, MN).

### Western Blot

The supernatant (1 ml) of cultured cells was collected in cryogenic vials and stored frozen at −80°C for 4 h. A rotational vacuum concentrator was used to lyophilize the samples. The lyophilized product was dissolved in 100 μl water and mixed with sodium dodecyl sulfate (SDS) loading buffer for Western blot using antibodies against cleaved IL-1β.

THP-1 differentiated macrophages lysates were prepared with RIPA lysis buffer (50 mM Tris, pH = 7.4, 150 mM NaCl, 1% Triton X-100, 1% sodium deoxycholate, and 0.1% SDS) with proteinase inhibitor cocktail, followed by the test of protein concentration with BCA assay (Takara, Japan) and mixed with SDS loading buffer.

Supernatants or cell lysate samples were electrophoresed in SDS-polyacrylamide gel electrophoresis gel (PAGE) and transferred onto nitrocellulose (NC) membrane (Millipore, MA, USA). NC membranes were blocked with 5% skimmed milk in TBS with 0.1% Tween-20 (TBST) and incubated with the antibody at 4°C overnight. After incubation with the HRP-conjugated secondary antibody, protein band was detected using SuperSignal West Pico Chemiluminescent Substrate Kit (Thermo Scientific, Rockford, IL) according to the manufacturer’s instructions. All the western-blot experiments were done at least twice.

### Immunofluorescence Assay

Monoclonal antibody TF2 was isolated using recombinant SFTSV nucleoprotein (NP) as a bait from a phage-display antibody library derived from the peripheral blood mononuclear cells of a patient that recovered from SFTS disease. Since only the sequence of the variable region was available, the full-length heavy and light chains were constructed by combining the variable region with the constant region of human IgG1. The full-length mAb TF2 was produced by cotransfection in HEK293T cells with heavy and light chains and purified by a protein A column and a Superdex 200 column.

The infected monocytes were fixed with 4% paraformaldehyde for 15 min. After washing with PBS, the cells were permeabilized with PBS containing 0.2% Triton X-100 for 10 min and blocked with PBS containing 5% BSA for another 1 h. The cells were incubated with anti-SFTSV NP mAb TF2 at 4°C overnight, followed by the incubation with FITC-conjugated anti-human IgG for 30 min. After washing, cells were incubated with 4,6-diamidino-2-phenylindole (DAPI) solution for 5 min and washed three times with PBS. The cells were analyzed with a fluorescence microscope (Olympus, Tokyo, Japan).

### ASC Oligomerization

ASC oligomerization was detected as previously reported (29). Macrophages were seeded in 6-well plates (2 × 10^6^ cells per well) and treated with different stimuli. The cells were pelleted by centrifugation and lysed by Pierce™ IP Lysis Buffer (Thermo) with PMSF and a protease inhibitor mixture. The cell lysates were then centrifuged at 5,000*×g* for 10 min at 4°C, and the pellets were washed twice with ice-cold PBS and resuspended in 500 μl of PBS. Next, the resuspended pellets were crosslinked with fresh DSS (4 mM) for 30 min and pelleted by centrifugation at 5,000*×g* for 10 min. The crosslinked pellets were resuspended in 40 μl of non-reducing SDS buffer, separated using 12% SDS-PAGE and immunoblotted using anti-human ASC antibodies.

### Statistical Analyses

The results were expressed as mean values (± standard deviations). Statistical analyses were performed using a two-tailed student t-test or one-way ANOVA (for multiple comparisons) followed by a Sidak, Tukey or Dunnett multiple comparison test as appropriate. GraphPad Prism 8 was used to generate all charts and statistical analyses, and Gaussian distribution and correction post-test of statistics analysis was performed. Any p-value less than or equal to 0.05 was considered statistically significant.

## Results

### SFTSV Infection Triggers NLRP3 Inflammasome Activation and IL-1β Secretion

**Figure 1:**
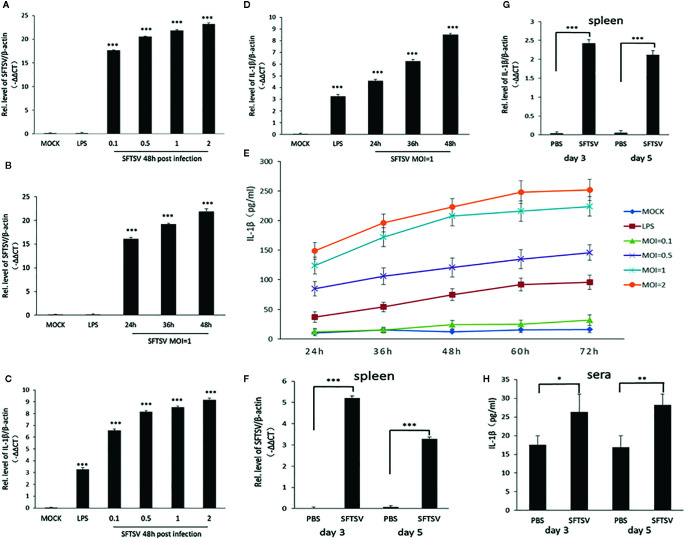
SFTSV infection induces the release of the pro-inflammatory cytokines interleukin-1β (IL-1β) in human PMBCs and C57/BL6 mice. **(A, B)** SFTSV viral RNA and β-actin mRNAs in human PBMCs were quantified by qPCR. **(C, D)** IL-1β and β-actin mRNAs in human PBMCs were quantified by qPCR. **(E)** Human PBMCs were infected with SFTSV at different MOIs (0.1, 0.5, 1, and 2) or treated with LPS. IL-1β levels were measured in the supernatants by enzyme-linked immunosorbent assay (ELISA). **(F)** SFTSV viral RNA and β-actin mRNAs in mice spleen were quantified by qPCR. **(G)** IL-1β and β-actin mRNAs in mice spleen were quantified by qPCR. **(H)** IL-1β levels in mice sera were determined by ELISA. Data are mean values ± SD derived from the samples collected in two times under identical conditions. *P<0.05, **P<0.01, ***P<0.001.

PBMCs were used as a cell model to determine the release of IL-1β during SFTSV infection, where PBMCs were infected at various MOIs (0.1, 0.5, 1, and 2). Cells treated with LPS were used as a positive control. SFTSV RNA was detected in infected PBMCs in 24, 36, and 48 h, which indicated that SFTSV can infect PBMCs and replicated well in the cells (**Figures 1A, B**). The mRNA expression of IL-1β, NLRP3, caspase-1, and ASC were stimulated by LPS and SFTSV (**Figures 1C, D**, **Figures S1A–F**). IL-1β level significantly increased in the supernatants of cells infected with SFTSV or cells treated with LPS than those of mock cells (**Figure 1E**). Next C57/BL6 mice were infected with SFTSV or mock infection, the viral RNA was detected, and the declined trend was observed from day 3 to day 5 in the spleen (**Figure 1F**). IL-1β mRNA expression in the spleen and IL-1β in the sera increased compared to the mock mice (**Figures 1G, H**), demonstrating that SFTSV induces IL-1β production and secretion.

**Figure 2:**
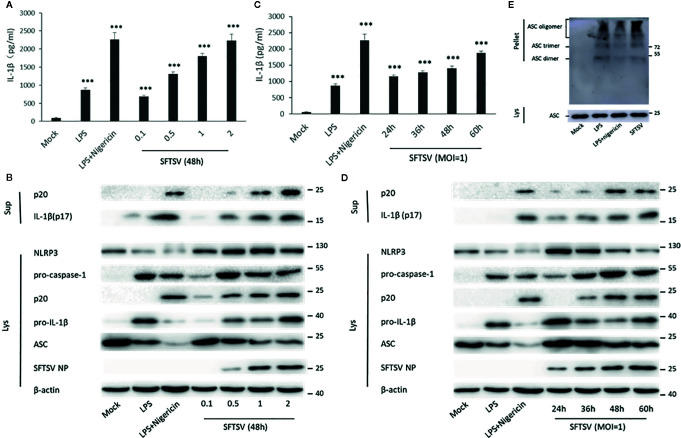
SFTSV infection triggers NLRP3 inflammasome activation and IL-1β production and secretion. **(A, B)** THP-1 macrophages were treated with LPS (1 μg/ml) for 4 h (or then 2 μM Nigericin for 30 min) or infected with SFTSV for 48 h at different MOIs (0.1, 0.5, 1, or 2). **(C, D)** THP-1 macrophages were treated with LPS (1 μg/ml) for 4 h (or then 2 μM Nigericin for 30 min) or infected with SFTSV at an MOI = 1 for 24, 36, 48, or 60 h. **(A, C)** IL-1β levels in the supernatant were determined by ELISA. **(B, D)** Mature IL-1β and cleaved caspase-1 in supernatants or NLRP3, pro-caspase-1, cleaved caspase-1 pro-IL-1β, ASC, SFTSV NP and *β*-actin in lysates were determined by Western blot. **(E)** THP-1 macrophages were treated with LPS (1 μg/ml) for 4 h (or then 2 μM Nigericin for 30 min) or infected with SFTSV at an MOI = 1 for 48 h. ASC oligomerization was determined by Western blot. Data are mean values ± SD derived from the samples collected in triplicate. ***P<0.001.

THP-1 differentiated macrophages were also applied to determine the effect on the IL-1β secretion during SFTSV infection. An anti-SFTSV NP monoclonal antibody TF2 was expressed and purified (**Figure S2**). SFTSV NP was detected in the macrophages, indicating that SFTSV replicated well in the cells (**Figure S3** and **S4**). Compared with the mock infection, pro-IL-1β in cell lysates, IL-1β and cleaved caspase-1 (p20) in supernatants were induced by LPS, LPS/Nigericin, and SFTSV (**Figures 2A–D**). In SFTSV infected cells, accumulation of NLRP3, pro-caspase-1, and cleaved caspase-1 was observed compared with the mock infection, and ASC dimer, trimer, and oligomer were detected in the infected cells (**Figures 2B, D, E**). In summary, we demonstrated that SFTSV infection could activate NLRP3 inflammasome and induce the production and secretion of IL-1β.

### SFTSV Infection Induced IL-1β Secretion Is Dependent on the Active Caspase-1 Formation

**Figure 3:**
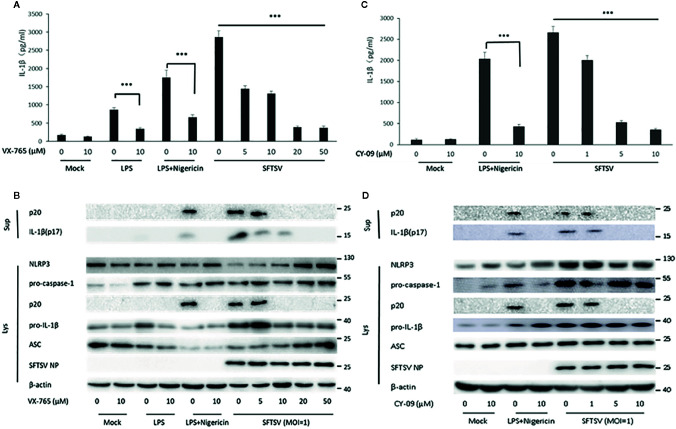
SFTSV infection induced IL-1β secretion is dependent on active caspase-1 and NLRP3 assembly. **(A, B)** THP-1 macrophages were treated with the caspase-1 inhibitor (VX-765) at indicated concentration for 1 h prior to infection. Then the cells were treated with LPS (1 μg/ml) for 4 h (or then 2 μM Nigericin for 30 min) or infected with SFTSV at an MOI = 1 for 48 h. **(C, D)** THP-1 macrophages were treated with the NLRP3 assembly inhibitor (CY-09) at indicated concentration for 1 h prior to infection. Then the cells were treated with LPS (1 μg/ml) for 4 h (then 2 μM Nigericin for 30 min) or infected with SFTSV at an MOI = 1 for 48 h. **(A, C)** IL-1β levels in the supernatant were determined by ELISA. **(B, D)** Mature IL-1β and cleaved caspase-1 in supernatants or NLRP3, pro-caspase-1, cleaved caspase-1 pro-IL-1β, ASC, SFTSV NP and *β*-actin in lysates were determined by Western blot. Data are mean values ± SD derived from samples collected in triplicate. ***P<0.001.

To determine the mechanism associated with the secretion of IL-1β upon SFTSV infection. We first assessed the role of caspase-1 in the SFTSV-induced IL-1β secretion. In THP-1 differentiated macrophages supernatants, IL-1β and cleaved caspase-1 level was significantly higher in the LPS/Nigericin or SFTSV-treated cells than mock infected cells (**Figures 2B, D**). LPS or LPS/Nigericin stimuli served as a positive control for stimulation of IL-1β secretion. To inhibit the proteinase activity of caspase-1, caspase-1 inhibitor VX-765 was added to the medium prior to and during SFTSV infection and LPS treatment. We observed a significant increase in the release of IL-1β in the supernatant of SFTSV infected cells, LPS, or LPS/Nigericin-treated cells compared with mock-infected cells on 48 h after infection. At the same time, IL-1β secretion stimulated by SFTSV infection or LPS/Nigericin was all repressed by inhibitor VX-765 on 48 h after infection (**Figures 3A, B**). The decrease of IL-1β level in the supernatants of the SFTSV infected macrophages was dose-dependent on VX-765 (**Figure 3B**). Collectively, these data demonstrated that active caspase-1 plays a dominant role in the IL-1β secretion induced by SFTSV infection.

### NLRP3 Inflammasome Is Involved in SFTSV Induced IL-1β Secretion

Previous studies have demonstrated that the IL-1β maturation and secretion are mediated by cleavage of caspase-1 under the control of the inflammasome, including NLRP3, NLRC4, and AIM2 inflammasome (13). Here, we focused on NLRP3 inflammasome, the best-characterized inflammasome involved in the maturation and secretion of IL-1β. To examine the role of NLRP3 inflammasome in the SFTSV induced release of IL-1β, NLRP3 assembly inhibitor CY-09 was added to the medium prior to and during SFTSV infection and LPS/Nigericin treatment. A significant increase of IL-1β was observed in the supernatant of SFTSV infected cells, or LPS/Nigericin-treated cells (**Figure 3C**). However, IL-1β and cleaved caspase-1 secretion stimulated by SFTSV infection or LPS/Nigericin were all inhibited by the inhibitor CY-09 (**Figure 3D**). And we also found that the decrease of IL-1β level in the supernatants of the SFTSV infected macrophages was dose-dependent on CY-09 (**Figures 3C, D**). The results demonstrated that NLRP3 assembly plays a crucial role in SFTSV infection induced IL-1β secretion.

**Figure 4:**
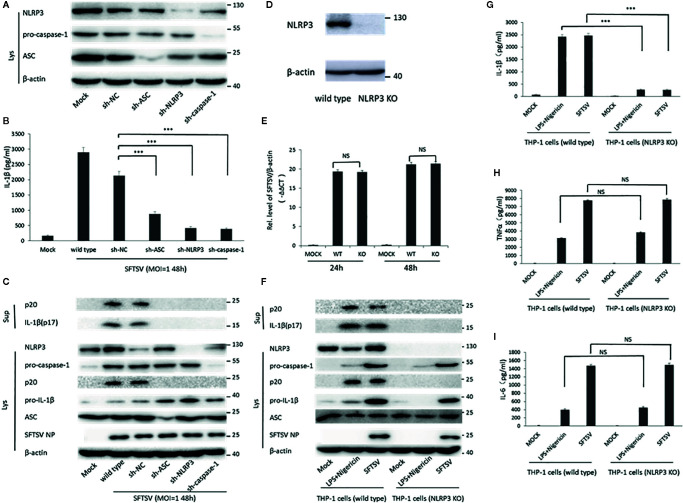
The role of NLRP3 inflammasome in the regulation of SFTSV induced IL-1β secretion. **(A)** THP-1 cells expressing shRNAs with the target at ASC, NLRP3, or pro-caspase-1 were lysed, and the targeted proteins were determined by Western blot at 3 days after lentiviral particles infection. THP-1 cells expressing shRNAs targeting ASC, NLRP3, or pro-caspase-1 were infected with SFTSV (MOI = 1) or mock infection for 48 h. **(B)** IL-1β levels in the supernatant were determined by ELISA. **(C, F)** Mature IL-1β and cleaved caspase-1 in supernatants or NLRP3, pro-caspase-1, cleaved caspase-1 pro-IL-1β, ASC, SFTSV NP and *β*-actin in lysates were determined by Western blot. **(D)** THP-1 cells (wild type and NLRP3 KO) were lysed, and the targeted proteins were determined by Western blot. **(E)** Differentiated THP-1 cells (wild type and NLRP3 KO) were infected with SFTSV (MOI = 1) or mock infection for 24 or 48 h. SFTSV viral RNA and *β*-actin mRNAs were quantified by qPCR. **(G–I)** Differentiated THP-1 cells (wild type and NLRP3 KO) were infected with SFTSV (MOI = 1) or treated with LPS/Nigericin or mock infection for 48 h. IL-1β, TNF-α and IL-6 levels in the supernatant were determined by ELISA. Data are mean values ± SD derived from the samples collected in triplicate. ***P < 0.001, NS, no significance.

Next, short hairpin RNA (shRNA) was used to knockdown the components of NLRP3 inflammasome (NLPR3, ASC, and pro-caspase-1). Western blot confirmed the knockdown of NLPR3, ASC, and pro-caspase-1 at protein level 3 days after lentivirus infection (**Figure 4A**). Wild-type THP-1 differentiated macrophages and target proteins knockdown cells were infected with SFTSV at MOI = 1 for 48 h. We found that mature IL-1β and cleaved caspase-1 were significantly lower in sh-NLRP3, sh-ASC, and sh-caspase-1 than the wild type and control macrophages in the supernatants (**Figures 4B, C**). While in the cell lysates, significant accumulation of pro-IL-1β was detected in the shRNAs’ knockdown cells compared with the wild type cells (**Figure 4C**). In all, the knockdown of NLRP3 inflammasome components attenuated the SFTSV induced IL-1β maturation and release.

Moreover, we screened NLRP3-knockout THP-1 cells by a CRISPR-Cas9 system and puromycin treatment (**Figure 4D**). The wild type and NLRP3 KO THP-1 cells were infected with SFTSV (MOI = 1) or stimulated with LPS/Nigericin or mock infection. After SFTSV infection and LPS/Nigericin stimulation, the knockout of NLPR3 was confirmed (**Figure 4F**). SFTSV replication was not affected by NLRP3 knockout (**Figures 4E, F**). IL-1β was observed with a significant decrease in the supernatant of SFTSV infected NLRP3 KO THP-1 cells 48 h after infection compared with the wild type THP-1 cells, while no significant change of TNF-α and IL-6 was detected in the NLRP3 KO cells compared with wild type cells (**Figures 4G–I**). The result was the same in the LPS/Nigericin stimulated wild type and NLRP3 KO THP-1 cells (**Figures 4G–I**). In summary, in SFTSV infection, IL-1β secretion was mainly regulated by NLRP3 inflammasome activation.

## Discussion

Inflammasomes are important components of the host innate immune system against pathogen infection (13). Previous studies have demonstrated that *mycobacterium tuberculosis* and *Candida albicans* were able to activate NLRC4 and NLRP3 inflammasomes (30–32), while Influenza virus, Zika virus, Enterovirus 71, and Hepatitis virus C could activate NLRP3 inflammasome to stimulate pro-inflammatory cytokine IL-1β maturation and secretion (17, 18, 20, 33). However, the interaction between inflammasome and SFTSV is not clear. Here, we demonstrated that SFTSV infection could also activate the NLRP3 inflammasome leading to the cleavage of caspase-1 and the release of IL-1β. Human PBMCs were infected with SFTSV, and SFTSV RNA was detected in the infected cells, indicating that SFTSV replicated well in the PBMCs (**Figures 1A, B**). SFTSV infection promoted the transcription of IL-1β and NLRP3 inflammasome components and the secretion of mature IL-1β (**Figures 1C, D**, **Figure S1**). To determine the role of NLRP3 inflammasome in SFTSV infection induced IL-1β secretion, we found that treated with NLRP3 assembly inhibitor, the knockdown and knockout of NLRP3 in the THP-1 macrophages led to the prohibition of SFTSV-induced secretion of IL-1β. The results demonstrated that the NLRP3 inflammasome is required for maturation and release of IL-1β. In addition, after SFTSV infection, IL-1β release significantly decreased in NLRP3 knockout THP-1 macrophages than wild type cells, but it was higher than mock infection in NLRP3 knockout macrophages (**Figure 4G**). That was probably because SFTSV infection could activate other inflammasomes except for NLRP3 inflammasome.

It has been shown that potassium efflux, calcium influx, mitochondrial reactive oxygen species, even viral RNA or RNA cleavage products participate in the NLRP3 inflammasome activation to induce caspase-1 activation and IL-1β maturation (13, 34, 35). Thus far, several models have been proposed to explain NLRP3 inflammasome activation by RNA viruses. First, viral proteins could directly interact with NLRP3 to facilitate the assembly of NLRP3 inflammasome complex. For example, Zika virus NS5 protein and EV71 3D protein could interact with NLRP3 directly to active NLRP3 inflammasome (17, 18, 21). Second, HCV core protein and p7 protein enhanced calcium influx and potassium efflux to drive the NLRP3 inflammasome activation and IL-1β release in macrophages (36, 37). In addition, the 2B proteins of EMCV and human rhinovirus triggered NLRP3 inflammasome activation by inducing Ca^2+^ flux from the ER and Golgi compartments (38, 39). When infected, SFTSV expressed four proteins, including nucleoprotein (NP), glycoprotein (Gn/Gc), RNA-dependent RNA polymerase (RdRp), and non-structural protein (NSs). Previous studies reported that SFTSV NSs played an important role in immune escape to facilitate SFTSV replication. NSs could form inclusion bodies in the cytoplasm and hijack host cell molecules like STAT1/2 and IRF7 into the inclusion bodies to inhibit the NF-*κ*B signal pathway activation (28, 40–43). We also expressed SFTSV single protein (RdRp, Gn/Gc, NP and NSs) in THP-1 macrophages, and stimulated the cells by LPS, but no significant increase of IL-1β in the supernatant compared with LPS treated alone (data not show). However, a study by Moriyama et al. reported that both wild-type NSs and the 21/23A mutant of SFTSV suppressed NLRP3 inflammasome-dependent IL-1β secretion (44). We thought NLRP3 inflammasome activation in SFTSV infection might be stimulated by calcium influx or potassium efflux. To detect the effect of calcium influx/potassium efflux stimulation, THP-1 cells were pretreated for 30 min with BAPTA-AM (a chelator of intracellular Ca2^+^ stores) (0, 2, 4,8 μM) or KCl (used to prevent potassium efflux) (0, 3.125, 6.25, 12.5 mM) before SFTSV infection. The results showed that the secretion of IL-1β decreased with the increases of concentrations of BAPTA-AM and KCl. These results indicated that calcium influx/potassium efflux stimulation did influence IL-1β secretion during SFTSV infection (**Figure S5**). Zhang et al. found that SFTSV infection or glycoprotein expression alone was sufficient to stimulate endoplasmic reticulum stress (45), which might result in the inflammasomes activation. It was reported that cleaved caspase-1 could activate GSDMD to form the functional GSDMD-N to induce cell pyroptosis. We found that SFTSV infection could induce mild pyroptosis in the THP-1 macrophages. IL-1β level significantly increased in the supernatants, while IL-18 level was a slight higher in SFTSV infected supernatants (**Figures S6A, B**). GSDMD mRNA was stimulated, and GSDMD-C (cleaved GADMD C domain) increased in SFTSV infection THP-1 cells compared with mock infection, which indicated that SFTSV could induce pyroptosis (**Figure S6C, D**). But cell damage or membranolysis did not appear at 24 h post SFTSV infection (**Figure S6E**). It was probably that a SFTSV protein could inhibit the process of pyroptosis in the infection. The details of how SFTSV stimulates the release of pro-inflammatory cytokines through the activation of the inflammasome should be further investigated, and it will provide strategies for the intervention of SFTSV infection, especially for the critical patients.

In conclusion, we revealed that SFTSV infection facilitated the maturation and secretion of pro-inflammatory cytokine IL-1β through NLRP3 inflammasome activation.

## Data Availability Statement

The raw data supporting the conclusions of this article will be made available by the authors, without undue reservation.

## Ethics Statement

The animal study was reviewed and approved by the ethics committee of the medical school, Wuhan University (2019YF2013), and was conducted in accordance with the guidelines for the protection of animal subjects. Written informed consent was obtained from the owners for the participation of their animals in this study.

## Author Contributions

Conceptualization, X-JY and J-WL. Methodology, RQ and Y-JJ. Software, RQ. Resources, J-WL, MC, and C-MZ. Data writing—original draft preparation, J-WL and MC. Writing—review and editing, X-JY. Project administration, X-JY. Funding acquisition, X-JY. All authors contributed to the article and approved the submitted version.

## Funding

This study was supported by the National Natural Science Funds of China (No.81971939 and 31570926). The funders had no role in the study design, data collection and analysis, decision to publish, or the preparation of the manuscript.

## Conflict of Interest

The authors declare that the research was conducted in the absence of any commercial or financial relationships that could be construed as a potential conflict of interest.

## Supplementary Material

Supplementary Figure 1 SFTSV infection triggers NLRP3 inflammasome components transcription in PBMCs. Human NLRP3, caspase-1, ASC and *β*-actin mRNAs were quantified by qPCR. **P* < 0.05, ***P* < 0.01, ****P* < 0.001.

Supplementary Figure 2 Monoclonal antibody TF2 purification and binding activity with SFTSV. The full-length mAb TF2 was produced in HEK293T cells and purified by Superdex 200 column (top). SFTSV NP was stained with mAb TF2 by IFA after infection for 48 h in THP-1 macrophages. Blue color shows the nucleus. (bottom).

Supplementary Figure 3 SFTSV replicates well in the THP-1 macrophages. THP-1 macrophages were infected with SFTSV at MOI = 1 for 24, 36, 48, or 60 h or mock infection. SFTSV NP was stained with mAb TF2 by IFA (green). Blue color shows the nucleus.

Supplementary Figure 4 SFTSV replicates well in the THP-1 macrophages. THP-1 macrophages were infected with SFTSV for 48 h at different MOIs (0.1, 0.5, 1, or 2) or mock infection. SFTSV NP was stained with mAb TF2 by IFA (green). Blue color shows the nucleus.

Supplementary Figure 5 Calcium influx and potassium efflux stimulation influence IL-1β secretion during SFTSV infection. THP-1 cells were pretreated with BAPTA-AM (0, 2, 4,8 μM) and KCl (0, 3.125, 6.25, 12.5 mM) 30 min before SFTSV infection. The secretion of IL-1β was detected by ELISA after 48 h post infection.

Supplementary Figure 6 SFTSV infection triggers pyroptosis in THP-1 macrophages. THP-1 macrophages were treated with LPS (1 μg/ml) for 4 h (or then 2 μM Nigericin for 30 min) or infected with SFTSV for 24 h at different MOIs (0, 1, or 2). **(A, B)** IL-1β and IL-18 levels in the supernatant were determined by ELISA. **(C)** Human GSMDM mRNA was quantified by qPCR. **(D)** GSDMD, GSDMD-C and *β*-actin in lysates were determined by Western blot. **(E)** Cell membranolysis was determined by CCK8.
