# Supplementary figures and images for: SFTSV Infection Induced Interleukin-1β Secretion Through NLRP3 Inflammasome Activation
Source: Front Immunol. 2021 Feb 23;12:595140. doi: 10.3389/fimmu.2021.595140 (PMC7940371; doi:10.3389/fimmu.2021.595140)

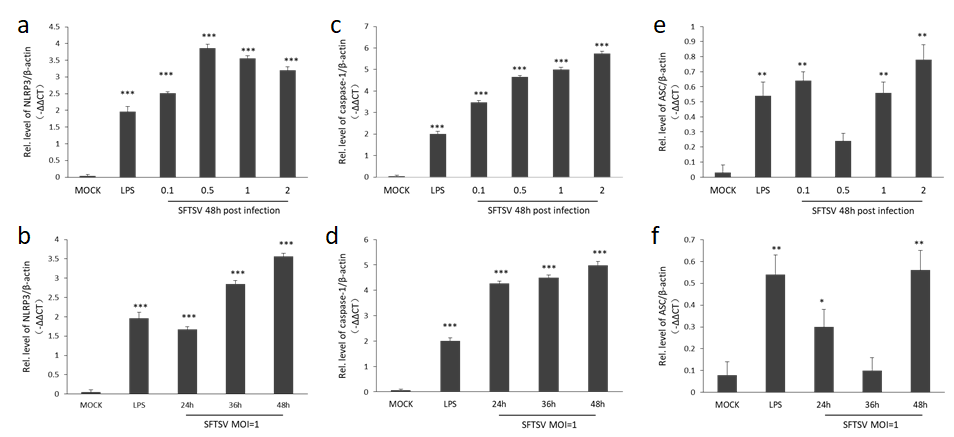

Supplement: Supplementary Figure 1 — SFTSV infection triggers NLRP3 inflammasome components transcription in PBMCs. Human NLRP3, caspase-1, ASC and β-actin mRNAs were quantified by qPCR. *P < 0.05, **P < 0.01, ***P < 0.001. [file Image_1.tif]

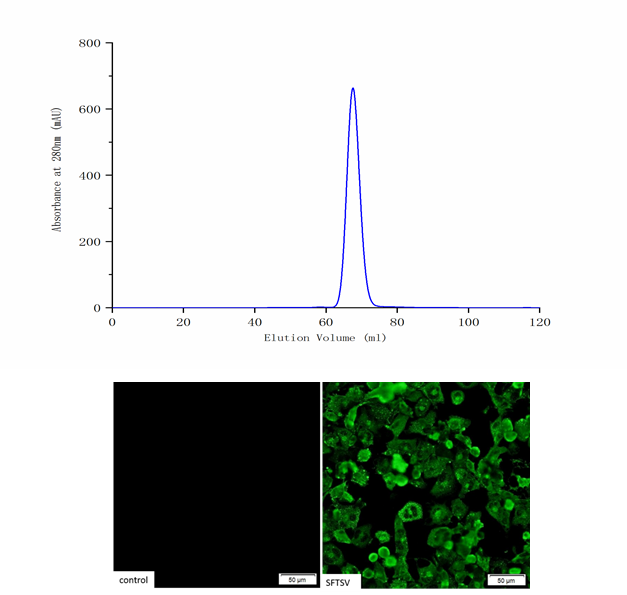

Supplement: Supplementary Figure 2 — Monoclonal antibody TF2 purification and binding activity with SFTSV. The full-length mAb TF2 was produced in HEK293T cells and purified by Superdex 200 column (top). SFTSV NP was stained with mAb TF2 by IFA after infection for 48 h in THP-1 macrophages. Blue color shows the nucleus. (bottom). [file Image_2.tif]

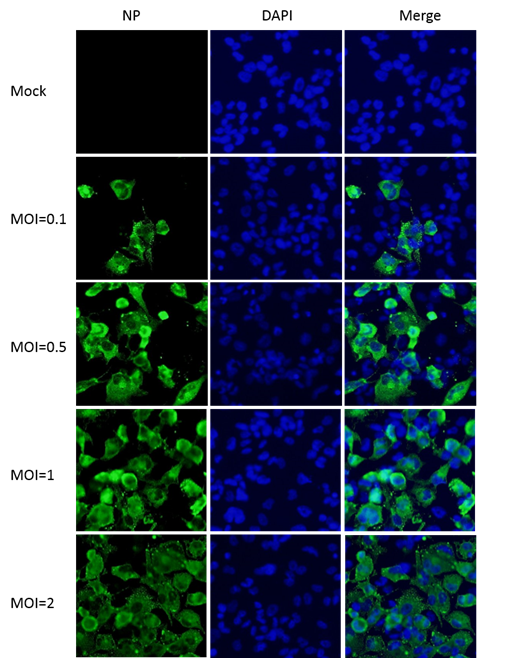

Supplement: Supplementary Figure 3 — SFTSV replicates well in the THP-1 macrophages. THP-1 macrophages were infected with SFTSV at MOI = 1 for 24, 36, 48, or 60 h or mock infection. SFTSV NP was stained with mAb TF2 by IFA (green). Blue color shows the nucleus. [file Image_3.tif]

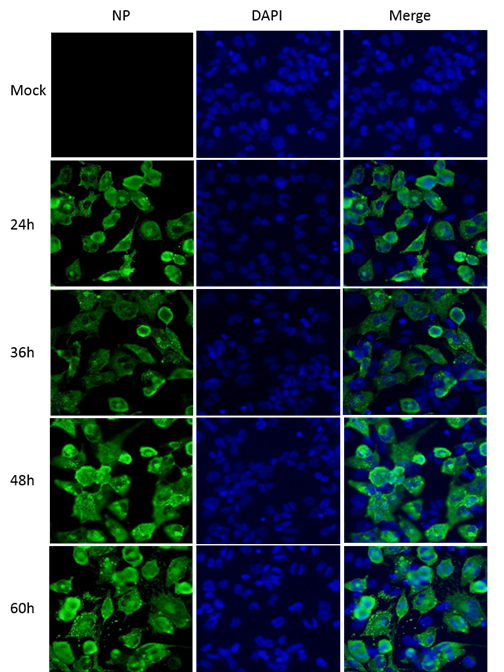

Supplement: Supplementary Figure 4 — SFTSV replicates well in the THP-1 macrophages. THP-1 macrophages were infected with SFTSV for 48 h at different MOIs (0.1, 0.5, 1, or 2) or mock infection. SFTSV NP was stained with mAb TF2 by IFA (green). Blue color shows the nucleus. [file Image_4.tif]

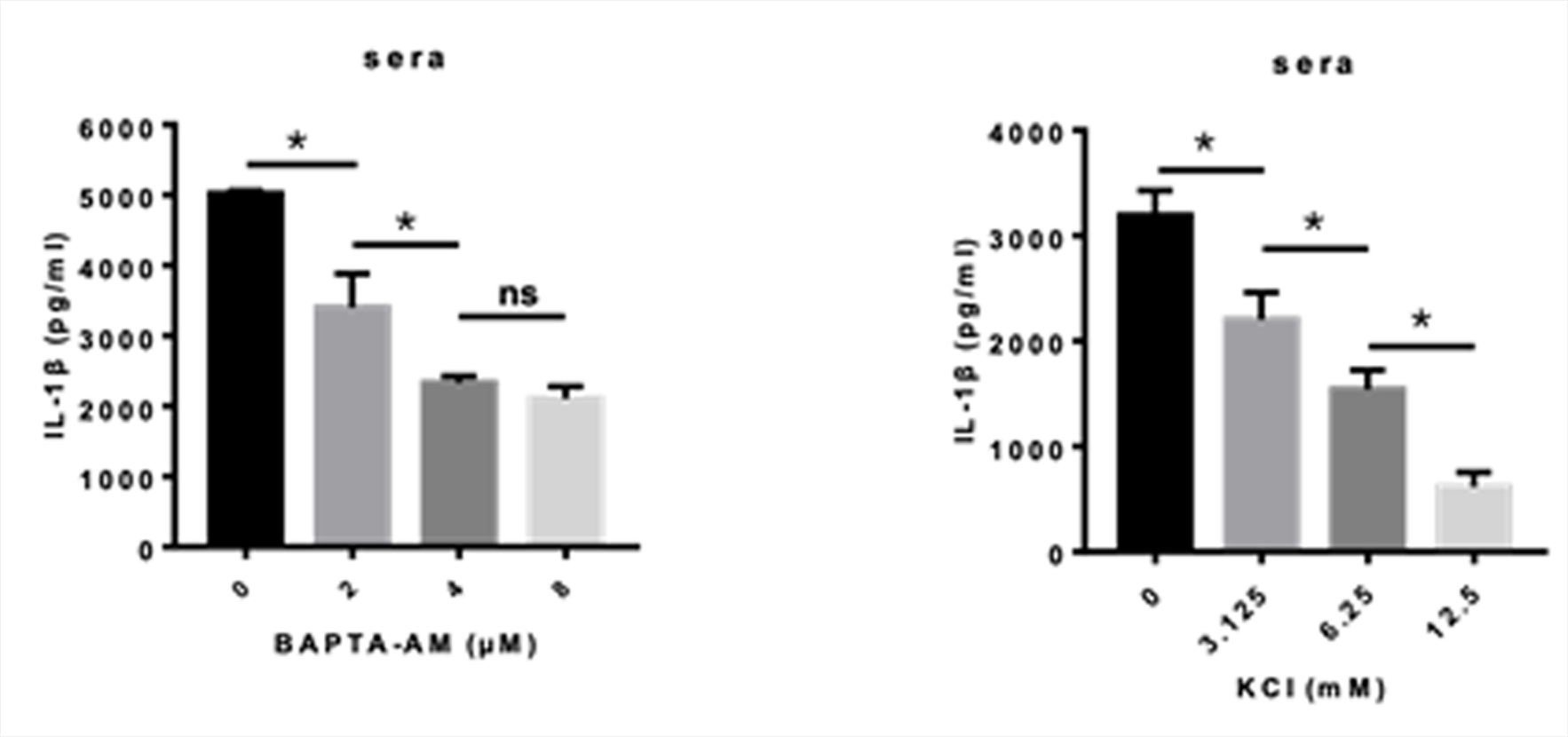

Supplement: Supplementary Figure 5 — Calcium influx and potassium efflux stimulation influence IL-1β secretion during SFTSV infection. THP-1 cells were pretreated with BAPTA-AM (0, 2, 4,8 μM) and KCl (0, 3.125, 6.25, 12.5 mM) 30 min before SFTSV infection. The secretion of IL-1β was detected by ELISA after 48 h post infection. [file Image_5.tif]

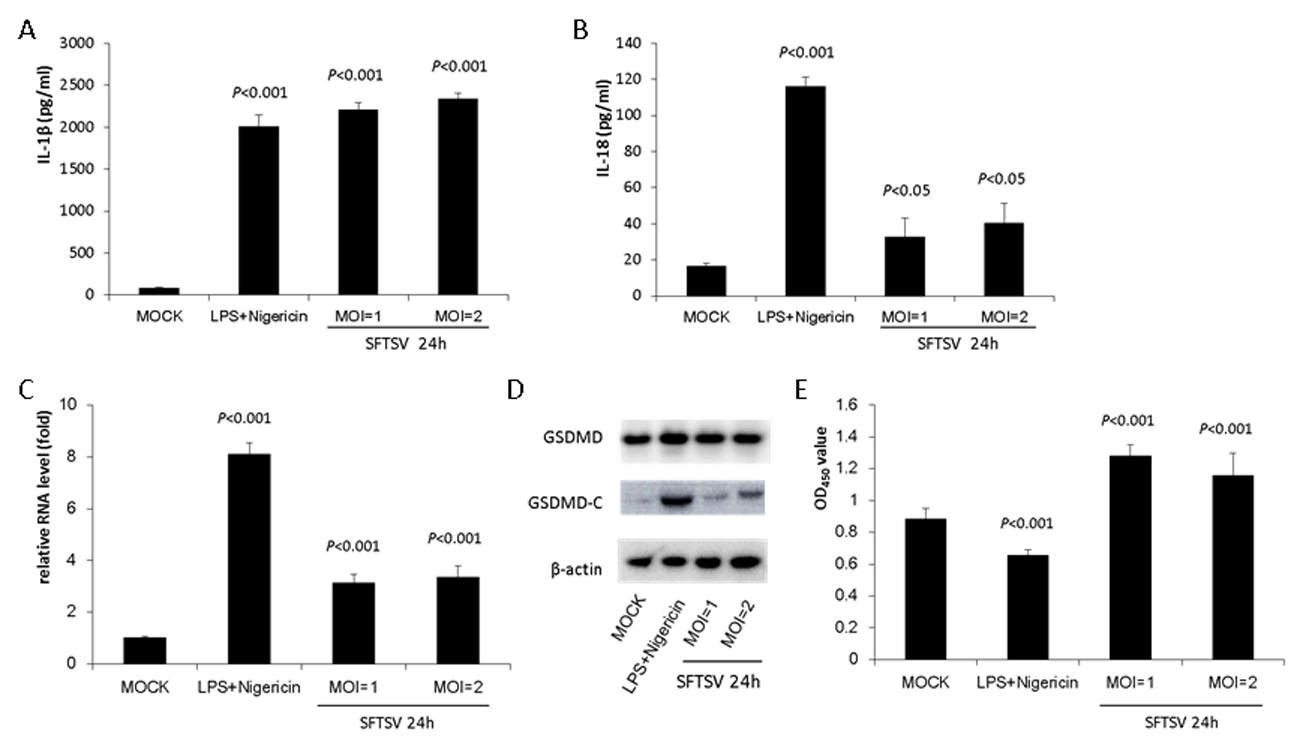

Supplement: Supplementary Figure 6 — SFTSV infection triggers pyroptosis in THP-1 macrophages. THP-1 macrophages were treated with LPS (1 μg/ml) for 4 h (or then 2 μM Nigericin for 30 min) or infected with SFTSV for 24 h at different MOIs (0, 1, or 2). (A, B) IL-1β and IL-18 levels in the supernatant were determined by ELISA. (C) Human GSMDM mRNA was quantified by qPCR. (D) GSDMD, GSDMD-C and β-actin in lysates were determined by Western blot. (E) Cell membranolysis was determined by CCK8. [file Image_6.tif]

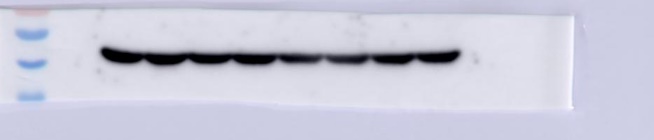
CY09 actin


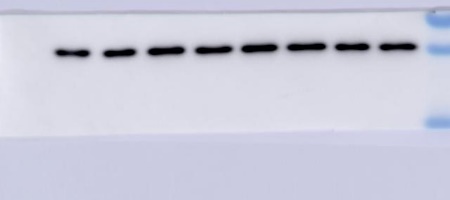
 CY09 ASC


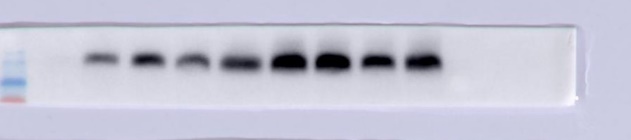
 CY09 NLRP3


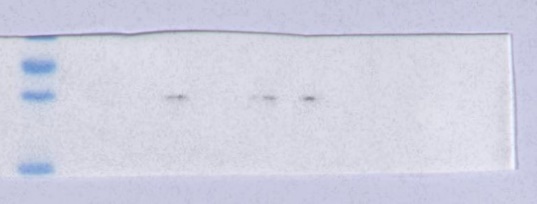
 CY09 p20 sup


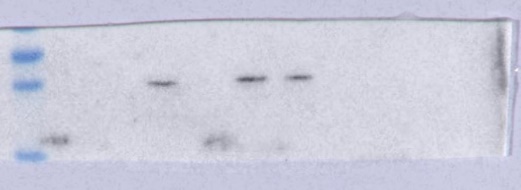
 CY09 p20


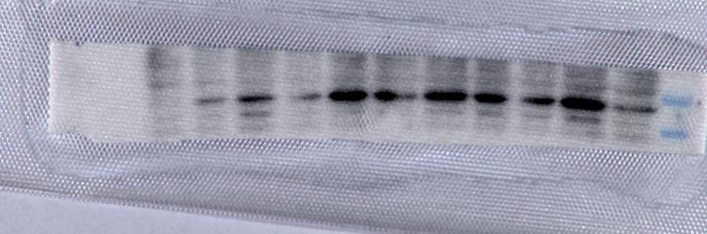
 CY09 pro-caspase1


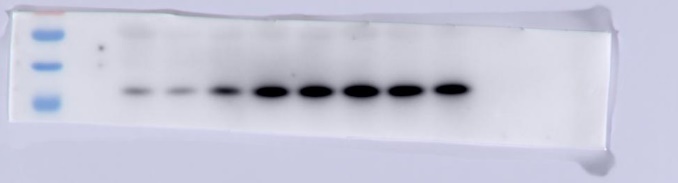
 CY09 proIL-1b


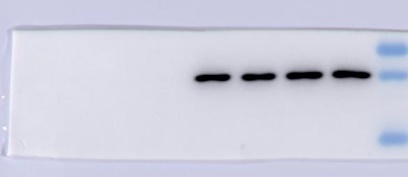
 CY09 SFTSV NP

Supplement: Supplementary file 7 [file Table_1.docx]

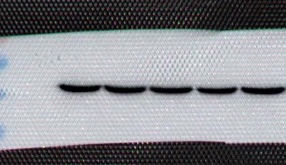
KD-shRNA actin


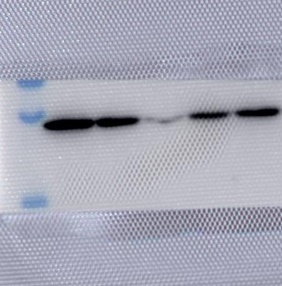
 KD-shRNA ASC


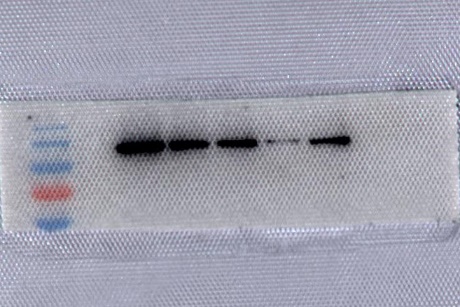
 KD-shRNA NLRP3

Supplement: Supplementary file 8 [file Table_2.docx]

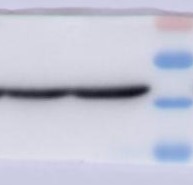
KO-sgRNA actin


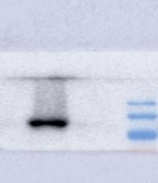
KO-sgRNA nlrp3

Supplement: Supplementary file 11 [file Table_5.docx]

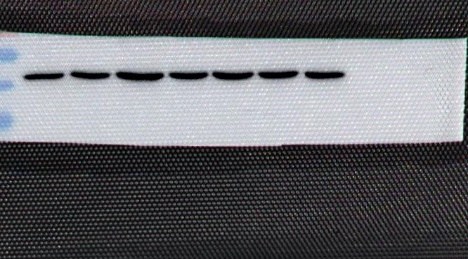
moi actin
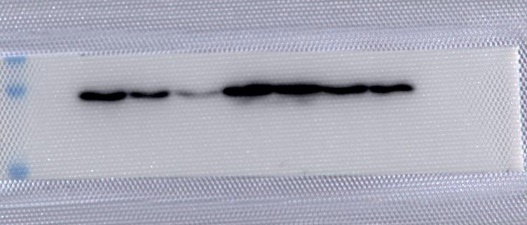
moi ASC


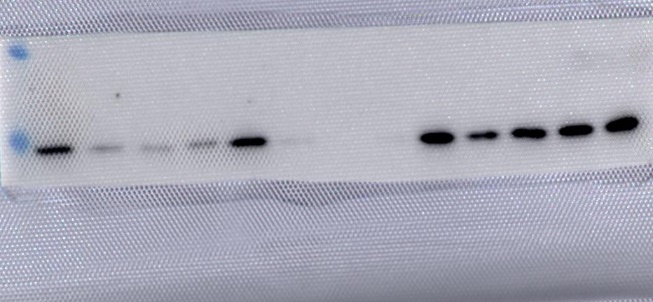
moi IL-1b


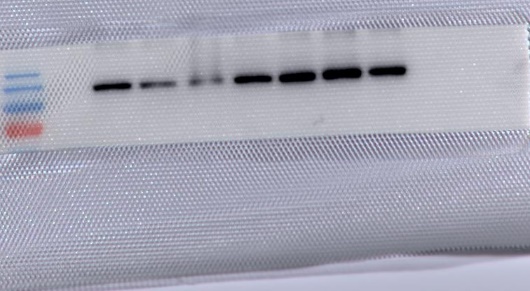
moi nlrp3


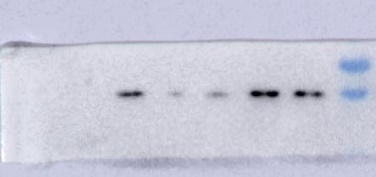
moi p20 sup


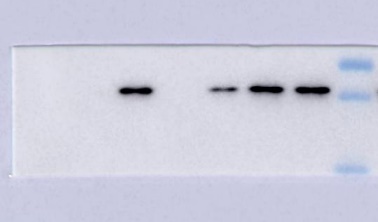
moi p20


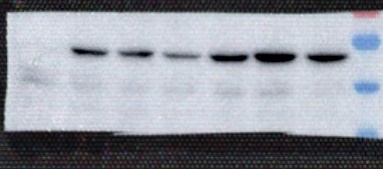
moi pro-casp1


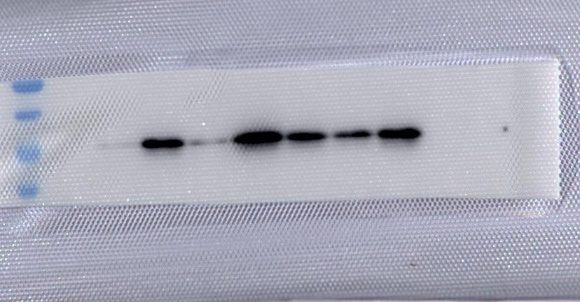
 moi pro-IL-1b


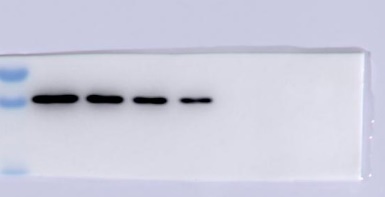
 moi SFTSV NP

Supplement: Supplementary file 12 [file Table_6.docx]

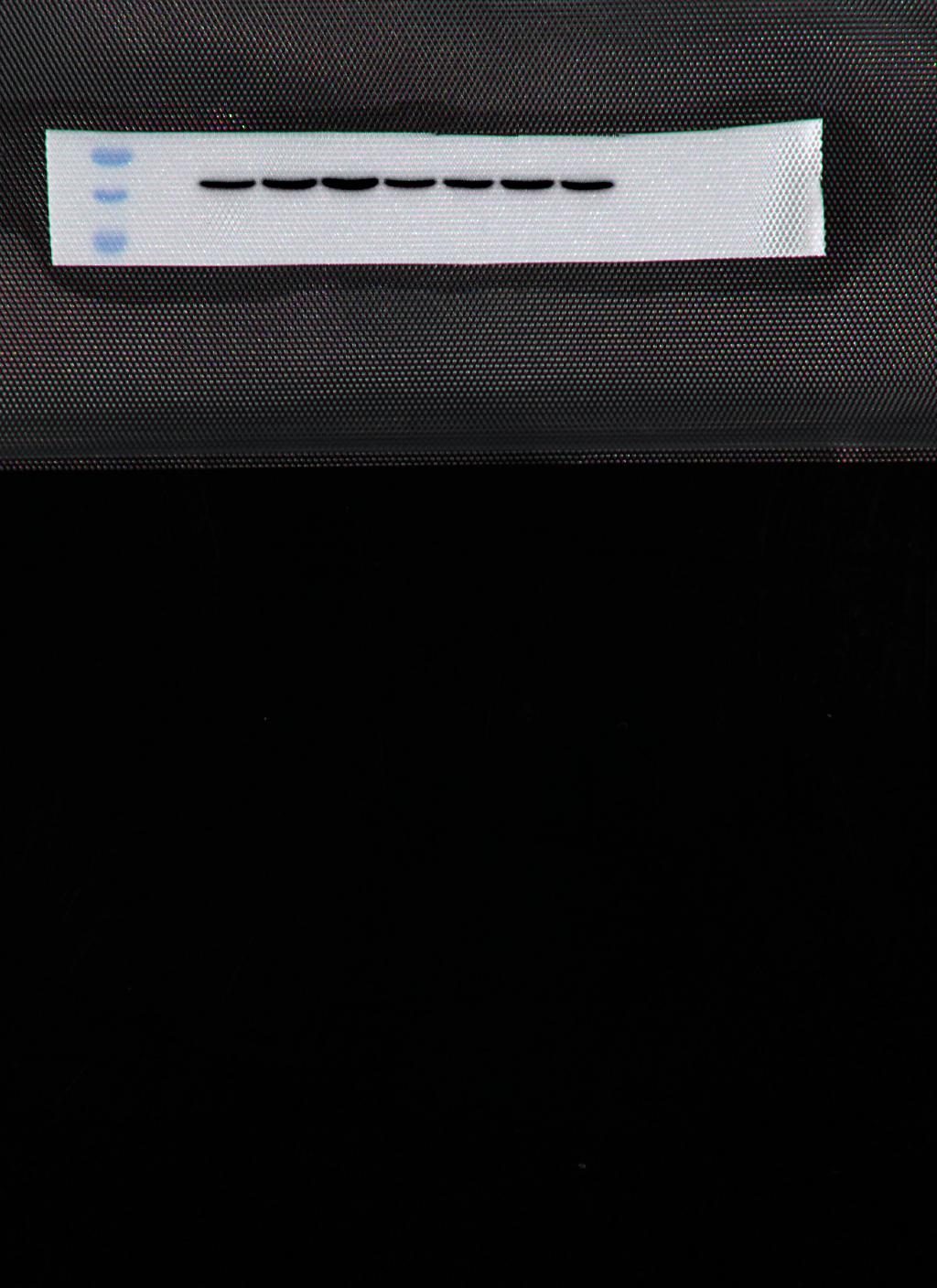
time actin


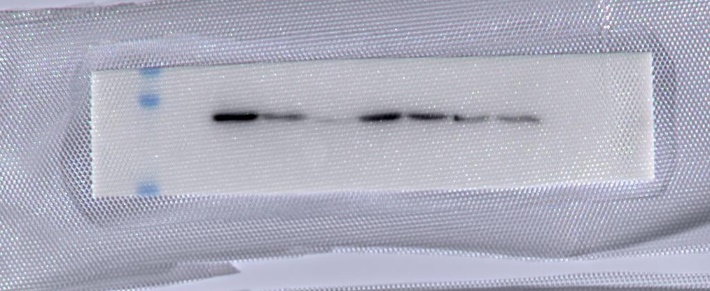
 time ASC


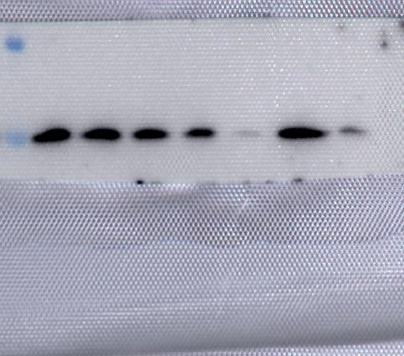
 time IL-1b


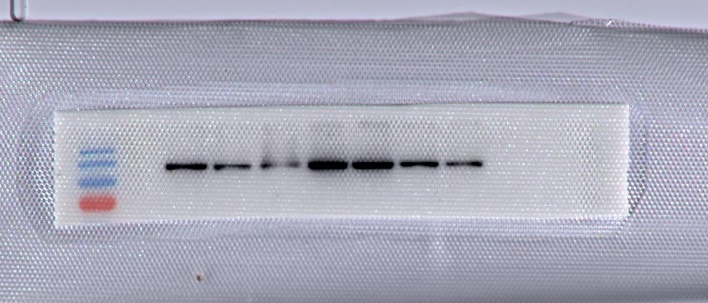
time nlrp3


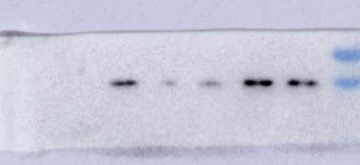
time p20 sup


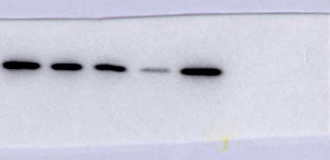
time p20


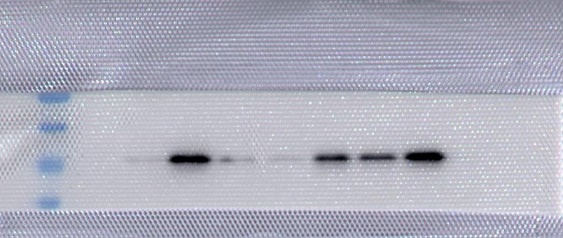
time pre-IL1b


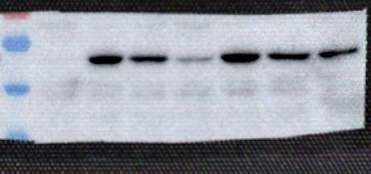
 time pro-casp1


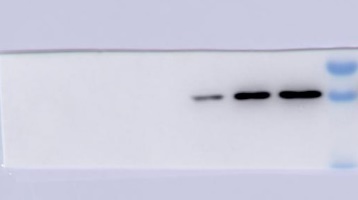
time SFTSV NP

Supplement: Supplementary file 13 [file Table_7.docx]

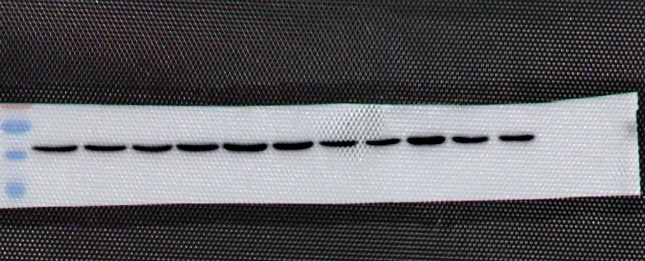
VX765 actin


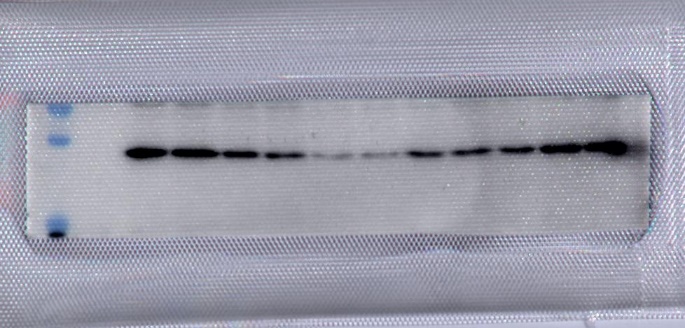
 VX765 ASC


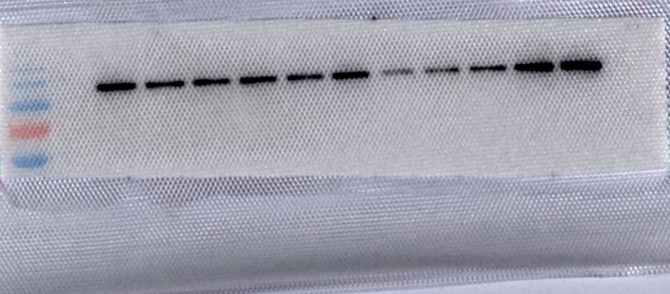
 VX765 nlrp3


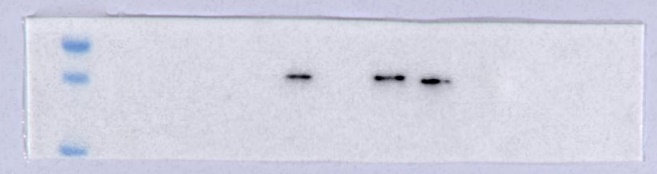
VX765 p20 sup


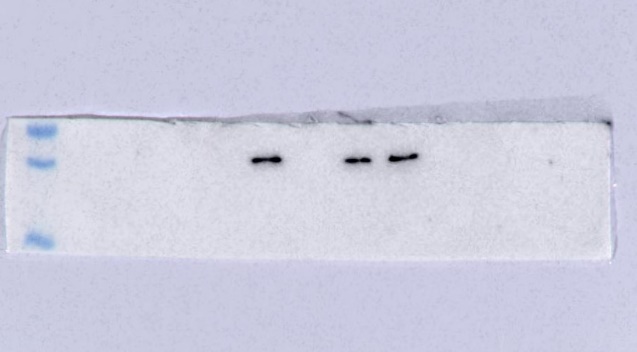
VX765 p20


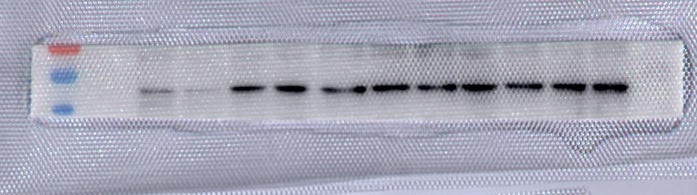
VX765 pro-casp1


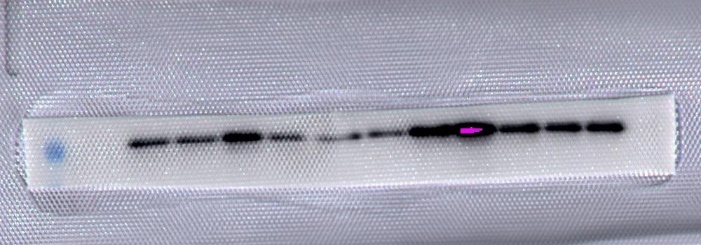
VX765 pro-IL1b


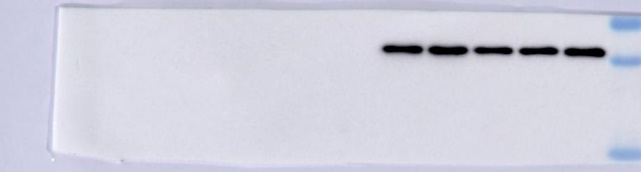
VX765 SFTSV NP

Supplement: Supplementary file 14 [file Table_8.docx]
