# Supplementary material for: SFTSV Infection Induced Interleukin-1β Secretion Through NLRP3 Inflammasome Activation
Source: Front Immunol. 2021 Feb 23;12:595140. doi: 10.3389/fimmu.2021.595140 (PMC7940371; doi:10.3389/fimmu.2021.595140)
Supplement: Supplementary file 9 [file Table_3.docx]

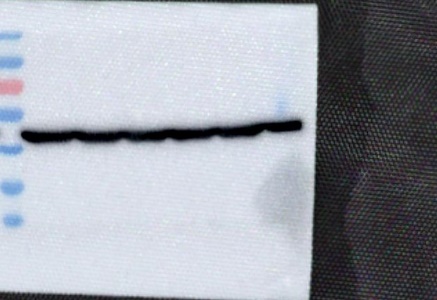
knockdown actin


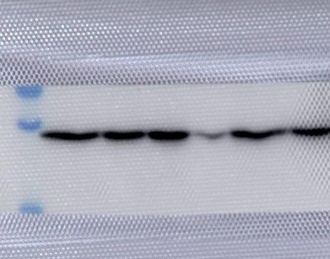
knockdown ASC


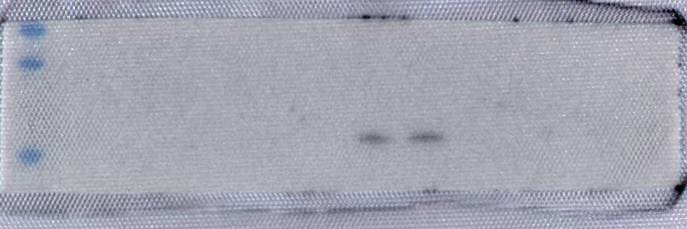
 knockdown IL-1b


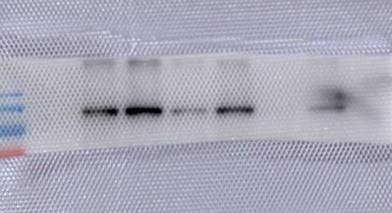
knockdown nlrp3


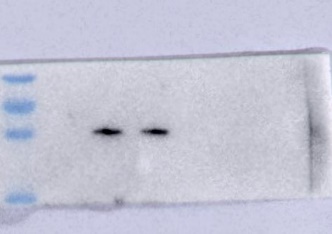
knockdown p20 sup


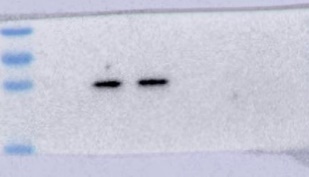
knockdown p20


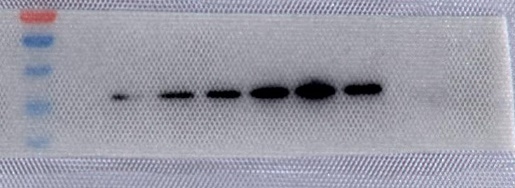
knockdown pre-IL1b


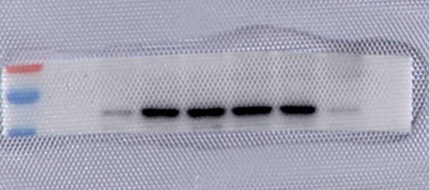
knockdown pro-caspase1


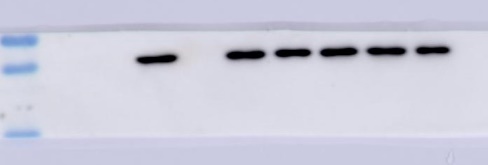
knockdown SFTSV NP
