# Supplementary material for: SFTSV Infection Induced Interleukin-1β Secretion Through NLRP3 Inflammasome Activation
Source: Front Immunol. 2021 Feb 23;12:595140. doi: 10.3389/fimmu.2021.595140 (PMC7940371; doi:10.3389/fimmu.2021.595140)
Supplement: Supplementary file 10 [file Table_4.docx]

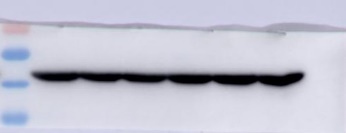
knockout actin


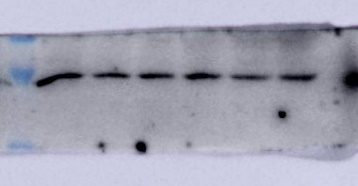
knockout ASC


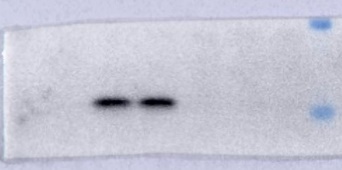
knockout IL-1b


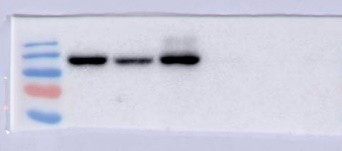
knockout nlrp3


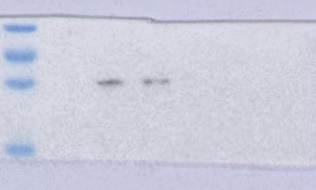
knockout p20 sup


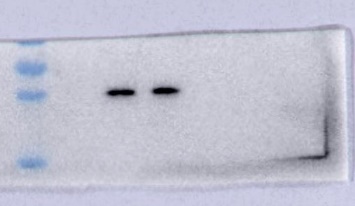
knockout p20


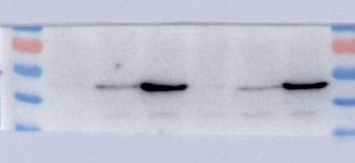
knockout procaspase-1


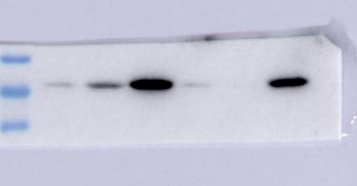
knockout proIL-1


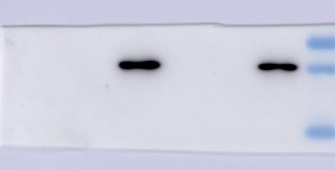
knockout SFTSV NP
